# Improvement of genome assembly completeness and identification of novel full-length protein-coding genes by RNA-seq in the giant panda genome

**DOI:** 10.1038/srep18019

**Published:** 2015-12-11

**Authors:** Meili Chen, Yibo Hu, Jingxing Liu, Qi Wu, Chenglin Zhang, Jun Yu, Jingfa Xiao, Fuwen Wei, Jiayan Wu

**Affiliations:** 1 CAS Key Laboratory of Genome Sciences and Information, Beijing Institute of Genomics, Chinese Academy of Sciences, Beijing, China; 2 Key Lab of Animal Ecology and Conservation Biology, Institute of Zoology, Chinese Academy of Sciences, Beijing, China; 3 Beijing Zoo 137 Xizhimen Outer St, Xicheng, Beijing, China

## Abstract

High-quality and complete gene models are the basis of whole genome analyses. The giant panda (*Ailuropoda melanoleuca*) genome was the first genome sequenced on the basis of solely short reads, but the genome annotation had lacked the support of transcriptomic evidence. In this study, we applied RNA-seq to globally improve the genome assembly completeness and to detect novel expressed transcripts in 12 tissues from giant pandas, by using a transcriptome reconstruction strategy that combined reference-based and *de novo* methods. Several aspects of genome assembly completeness in the transcribed regions were effectively improved by the *de novo* assembled transcripts, including genome scaffolding, the detection of small-size assembly errors, the extension of scaffold/contig boundaries, and gap closure. Through expression and homology validation, we detected three groups of novel full-length protein-coding genes. A total of 12.62% of the novel protein-coding genes were validated by proteomic data. GO annotation analysis showed that some of the novel protein-coding genes were involved in pigmentation, anatomical structure formation and reproduction, which might be related to the development and evolution of the black-white pelage, pseudo-thumb and delayed embryonic implantation of giant pandas. The updated genome annotation will help further giant panda studies from both structural and functional perspectives.

The giant panda (*Ailuropoda melanoleuca*) is a highly endangered mammal that has attracted worldwide conservation efforts for decades. Beyond their endangered status, many distinctive traits of giant pandas have attracted the attention of geneticists and evolutionary biologists, such as their black-white pelage, pseudo-thumb, specialized bamboo diet, low offspring-to-mother body weight ratio, and delayed embryonic implantation^1^. However, the genetic mechanisms governing these distinctive traits remain largely unknown. Insights into these problems will not only contribute to genetics and evolutionary biology but also facilitate the conservation of this rare and iconic species.

The giant panda whole genome was sequenced with next-generation sequencing technology and was the first *de novo* assembled genome based solely on short reads^2^. A gene set of 23,408 genes was annotated from the giant panda genome on the basis of a homology search with human and dog genes and *ab initio* methods^3^. The panda whole genome sequence provides an unprecedented opportunity to elucidate the panda’s biology and evolution. For example, genome sequence analysis found that the umami receptor gene *T1R1* has become a pseudogene due to frame-shift mutations and that cellulase-encoding genes do not exist in the giant panda genome^2^. The development of the whole genome sequence has facilitated the application of population genomics and meta-genomics in giant pandas, thereby providing deep insights into their population history, genome-scale evolutionary adaptation and the ability of their gut microbiome to degrade bamboo cellulose and hemi-cellulose^4^,^5^. Although the quantity of the annotated panda genes is comparable to that of other well-annotated mammalian genomes, short-read assembly inevitably causes trivial fragments and produces some gene gaps and missing UTRs. Moreover, the predicted gene models for the giant panda lack the support of transcriptomic data. A number of novel transcripts have been identified through transcriptome analysis in many model organisms with well-annotated genomes^6^,^7^,^8^,^9^, which emphasizes the complexity underlying genome annotation. Transcriptomic analysis of the genome of the giant panda (which is a non-model organism) should yield similar results.

Many genes may not be detected by homology search and *ab initio* methods alone^6^,^7^,^8^,^9^,^10^,^11^,^12^. RNA-seq technology based on next-generation sequencing has distinct advantages over classic microarray and serial gene expression analysis. RNA-seq not only detects and quantifies low-abundance transcripts but, more importantly, also identifies novel transcripts, alternative splicing and chimeric transcripts^13^,^14^,^15^. Using RNA-seq transcriptomic data to annotate a genome is an effective supplement to the traditional genome annotation method. Here, we reconstructed transcripts from the RNA-seq transcriptomic data of 12 giant panda tissues to verify the predicted gene models, fill gaps and boundaries, identify novel protein-coding transcripts, and improve the annotation of the panda genome. These findings will facilitate new insights into the genetics and evolutionary biology of this high-profile species.

## Results

### Sequencing and mapping of panda transcriptomes

We generated approximately 11.81 million and 25.88 million 101-nt paired-end reads for skeletal muscle and one skin sample, respectively, and approximately 40 million 80/100-nt paired-end reads for each of the other ten sampled giant panda tissues. After the filtering process, high-quality reads were mapped to the giant panda draft genome. As a result, 34.89% to 60.27% of the reads were mapped to known gene regions (details shown in Table S1). Based on the ailMel v1.62 gene models, 6.06–26.08% of the mappable reads were located on pure introns. Notably, 2.41–10.15% of the mappable reads were mapped to annotated intergenic regions (minus the 5 kb upstream and downstream of a gene), and 1.10–26.66% of the reads were located in scaffolds that contained no gene information. These results suggest that many novel transcribed loci failed to be annotated under the current computational annotation system.

### Transcriptome reconstruction

Based on the coverage information of the mappable splice-reads and read-pair links, Cufflinks^12^ was used to assemble the transcribed fragments into transcripts. All of the assembly results from the 12 tissue transcriptomes were merged into a 135,524-transcript set and a 90,218-transcribed loci set. The median transcript counts and transcribed loci counts by Trinity for the 12 tissue transcriptomes were 44,973 and 40,557, respectively.

### Improvement of genome assembly completeness

**Figure 1:**
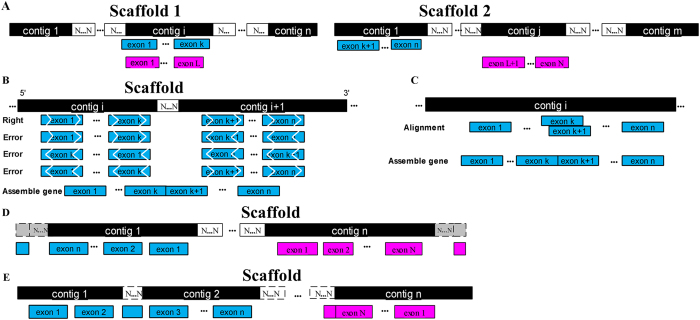
Diagram of the improvement in genome assembly completeness. (**A**) Scaffolding improvement; (**B**) Scaffolding inconsistencies; (**C**) Nest assembly errors; (**D**) Boundary extensions; (**E**) Gap closure.

The 656,239 Trinity-assembled transcripts^16^ were divided into three sets using TGNet: unaligned transcripts, aligned transcripts located within one scaffold, and aligned transcripts located within multiple scaffolds (Table S2). Of these transcripts, 184 showed 130 inconsistencies concerning the contig connection order or contig connection direction. As stated in the Methods section, the completeness of the panda genome assembly was improved in five ways as follows (Fig. 1): (1) In total, 7,438 transcripts were located in multiple scaffolds involving 2,106 scaffolds with 2,317 connections (Table 1). Of these connections, 741 were adjacent connections that could be used to improve scaffolding; another 79 were merging connections, suggesting a contig/scaffold had fallen into a gap region within one scaffold and enabling the improvement of inner scaffolding within a scaffold. Finally 1,503 connections indicated likely inconsistent scaffolding. (2) An inconsistent strand-orientation alignment implied mis-assembly. In total, 86 assembly errors in the ordinal split-alignment results and 84 errors in the reverse order split-alignment results were detected. (3) The locations of 14 transcripts indicated that the segments were nested, which implied the assembly of repeat-unit loss. (4) Additionally, 829 transcripts were located at scaffold boundaries, which allowed the extension of 279 scaffold boundaries up to 22,015-bp in coding-region length. (5) Last, 83,633 transcripts allowed the recovery of coding regions that fell into scaffold gap regions involving 17,586 gap regions in 2,403 scaffolds. Of these gap regions, 243,354-bp in length were filled with these transcript segments, and 6.64% of the total filled gap length was exactly matched to the predicted gap length.

The improved genome assembly region features and sequences are shown in Supplementary Note 1. The summary statistics for the original genome assembly and the improved genome assembly are shown in Table S3. After improvement using *de novo* assembled transcripts, the contig count reduced from 200,593 to 197,637. Contig N50 increased from 39,886-bp to 41,190-bp, and contig N90 increased from 9,848-bp to 10,081-bp. Genome assembly improvement was expected only in transcribed regions. To validate the genome assembly improvement, we calculated read pair mapping statistics of the selected whole genome sequencing data that were mapped to the original and improved genome sequences using Bowtie2. An additional 3,999 (0.01%) pairs were mapped to the improved genome assembly compared to the original genome assembly (Table S4). The mappable read pair count was slightly increased, which showed a little improvement based on the paired-end read alignment results.

### Identification of novel transcripts

**Figure 2:**
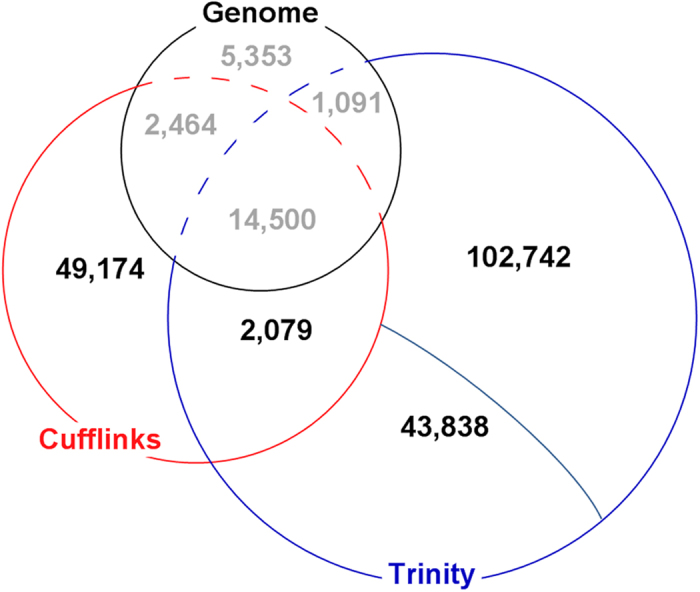
Comparison of the Cufflinks- and Trinity-assembled transcripts of the giant panda with known gene models. “Genome” represents known gene models from the Ensembl automated annotation system. In total, 43,838 Trinity-assembled transcripts unaligned back to the giant panda draft genome. In total, 102,742 Trinity-assembled transcripts were located to scaffolds that did not cover any known gene models.

The Trinity-assembled transcripts were classified into two types on the basis of the BLAT^17^ alignment results: unalignment and successful-alignment. The total of 85,894 unaligned transcripts indicated missing assembly in the draft genome and were defined as raw, reference-free candidate novel transcripts. According to sequence identity and sequence coverage information, the unaligned transcripts were clustered into 43,840 sequence clusters by cd-hit^18^. Then, 43,840 representative transcripts^10^ were selected out of these sequence clusters. Two representative transcripts were filtered out because they generated hits in the non-coding RNA (ncRNA) database^19^. As a result, 43,838 *de novo* assembled transcripts that had no proper genome position were selected as reference-free candidate novel genes for further validation (Fig. 2). The successful-alignment transcripts were clustered based on their overlapping genome locations. Transcripts that fell into repeat regions or known gene models were filtered out. As a result, 105,242 successful-alignment transcripts were selected as reference-dependent candidate novel genes (Fig. 2).

Cufflinks-assembled transcripts with an expression abundance <1 FPKM were filtered out. Additionally, transcripts that matched known gene models and fell into repeat regions were removed. As a result, 51,347 representative transcripts were selected as reference-dependent candidate novel genes. All reference-dependent candidate novel transcripts defined by Trinity and Cufflinks were clustered to remove the repetitive definition of novel genes. In total, 2,173 candidate novel transcripts from Cufflinks overlapped with 2,500 candidate novel transcripts from Trinity; consequently, 2,079 representative transcripts were selected based on the clustering results. Finally, 49,174 Cufflinks-defined transcripts, 102,742 Trinity-defined successful-alignment transcripts, 43,838 Trinity-defined unaligned transcripts, and 2,079 shared transcripts were identified as candidate novel genes. In total, 197,833 candidate novel genes were selected for further validation (pipeline shown in Supplementary Fig. S1).

### Validation of candidate novel protein-coding genes and gene function annotation

The Augustus^20^ prediction showed that 24,762 of the 197,833 transcripts contained 30,624 predicted open reading frames (ORFs). A total of 2,102 predicted ORFs were removed because they fell into known gene models or repeat regions. We used homology information to validate 28,522 potential novel protein-coding genes (pipeline shown in Supplementary Fig. S1). BLAST was used to search for sequences homologous to these transcripts. After filtering (see filtering criteria in the Methods section), we obtained three groups of ‘novel protein-coding genes’^21^: (1) 551 (1.93%) *homology-based genes* that were similar to known proteins in the nr database and known cDNA sequences in the nt database; (2) 6,290 (22.03%) *unknown genes* that were similar to EST sequences in dbEST but had no protein or cDNA homology information; and (3) 12,575 (44.09%) *hypothetical genes* that had a complete ORF but no known homologs. An InterProScan functional-domain search showed that 409 (74.23%), 5,112 (81.27%), and 7,981 (63.47%) genes in the homology-based, unknown, and hypothetical gene groups, respectively, had protein signature hits.

**Figure 3:**
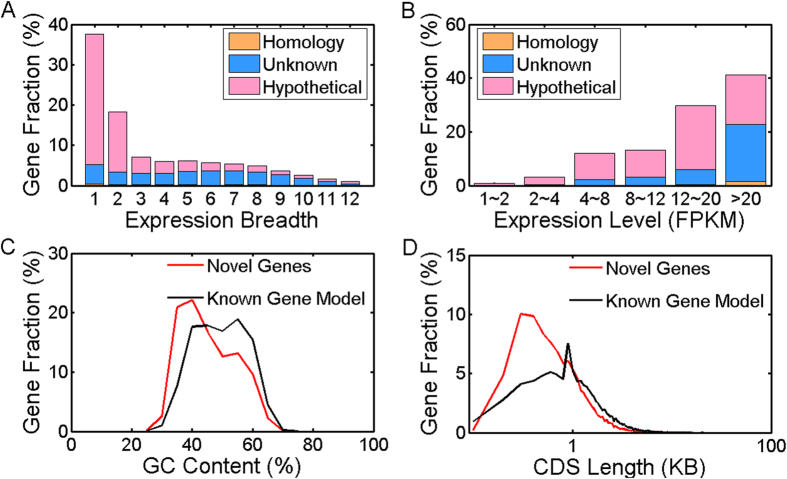
Expression pattern analysis of the giant panda novel protein-coding genes. (**A**) Expression breadth of the Trinity-defined novel protein-coding genes; (**B**) Expression distribution of the Trinity-defined novel protein-coding genes; (**C**) A comparison of the GC content distributions between all the novel protein-coding genes and the known gene models (step size was 5%) of the giant panda; (**D**) A comparison of the CDS length distribution between all novel protein-coding genes and the known gene models (step size was 0.1 KB) of the giant panda.

**Figure 4:**
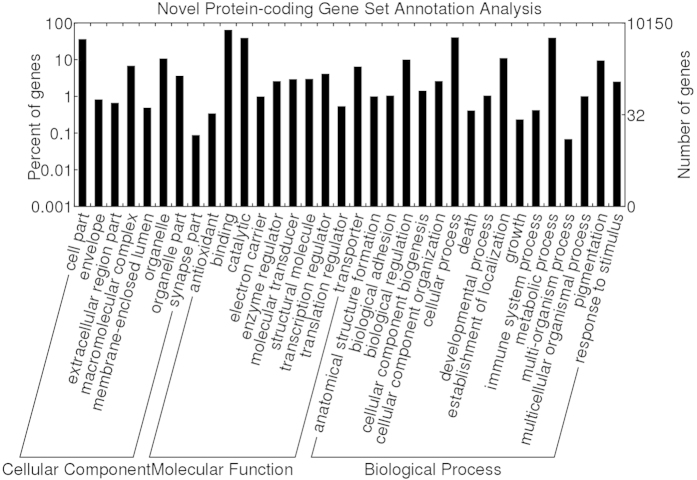
GO functional annotation analysis of the giant panda novel protein-coding genes.

These novel protein-coding genes primarily originated from highly expressed tissue-specific genes (Fig. 3A,B). The novel protein-coding genes had a slightly lower GC content compared with the known gene models (Fig. 3C). However, a two-tailed Wilcoxon rank sum test (p value ≈ 0.91) indicated that no significant difference existed between the 19,416 novel protein-coding genes and 21,122 known protein-coding gene models. The CDS lengths of the novel protein-coding genes predominantly ranged from between 200 to 800-bp, and the peak value of the CDS length distribution was approximately 350-bp. The median values were both 800-bp, but their distributions between the 19,416 novel protein-coding genes and 21,122 known protein-coding gene models were significantly different, as determined by a two-tailed Wilcoxon rank sum test (p value ≈ 0). The gene fraction of shorter novel genes was larger than that of the known gene models, whereas the gene fraction of larger novel genes was smaller than that of the known gene models (Fig. 3D). The shorter-gene bias for the novel genes inferred from the RNA-seq data is a common phenomenon that has been shown in bovines, rice and *Aspergillus oryzae*^7^,^22^,^23^. GO annotation analysis of the novel protein-coding genes showed that most genes encoded cell and organelle part components at the cellular component level. Moreover, these genes were primarily related to catalytic, binding, transporter and structural molecular activity at the molecular function level, and these genes were mainly involved in biological regulation, metabolic processes, cellular processes, anatomical structure formation, pigmentation and reproduction at the biological process level (Fig. 4).

### Proteomic validation of the novel protein-coding genes

Peptide hits in the novel protein-coding gene set were obtained after a BioWorks filtering analysis. A total of 12,043 peptide hits were detected to 1,691 novel protein-coding genes and 1,884 known gene models. Among the 10,962 Trinity-defined novel protein-coding genes expressed in the five selected tissues, 1,383 had peptide hits. The validation percentage of the novel protein-coding genes in the five selected tissues was approximately 12.62%. Among the 18,662 known gene models expressed in the five selected tissues, 1,882 had peptide hits (10.08%). The rates at which genes were validated in other species by proteomics were as follows: 23.91% in Planaria from eight worm whole transcriptomes^24^; 34.00% from mouse liver tissue^25^; and 37.70% for various newt tissues samples^26^. The proteomics validation results of the three types of novel protein-coding genes defined by Trinity from the five selected tissues were as follows: (1) 56 of 342 *homology-based genes* (16.37%) yielded peptide hits; (2) 780 of 5,407 *unknown genes* (14.43%) yielded peptide hits; and (3) 547 of 5,213 *hypothetical genes* (10.49%) yielded peptide hits (Table 2). Additionally, 1,400 peptides yielded hits to only 409 human and 170 dog known genes that were undetected in our identification of novel protein-coding genes. The undetected genes might originate from weakly expressed genes or partial genes that were filtered out in our identification pipeline. GO annotation analysis of the homology-based novel protein-coding genes that validated by proteome resulted in categories similar to those of the novel genes (Supplementary Fig. S2).

## Discussion

The giant panda genome is the first large mammalian animal genome built by *de novo* assembly using Illumina sequencing short reads alone^2^. In total, 12.08% of the draft genome sequences were inner gaps, and the Ensembl automated genome annotation system predicted 23,408 genes^3^. In this study, the reference-based transcriptome assembly by Cufflinks indicated that the transcriptomic data from the 12 tissues verified 77.13% of the known gene models. However, 5,353 gene models (22.87%), 30,027 exons (15.21%), and 25,869 introns (15.02%) were not covered by the transcriptomic data. The read alignment results indicated that fewer than half of the mappable reads were located in known gene regions. The proportion of reads covered by the known gene models in the giant panda transcriptome was much less than that of other animals. For example, the human brain transcriptome covers 86% of human genes^9^, three mouse transcriptomes cover 94.12% of mouse exons^8^, and 14 lizard deep-transcriptomes cover up to 97.36% of the corresponding Ensembl gene models^11^. Moreover, the unaligned transcripts in the giant panda were much more numerous than in other studies. Of the *de novo* assembled transcripts, 13.71% unaligned to the giant panda draft genome. In contrast, *de novo* assembled transcripts with unalignment in the lizard account for only 5.99% of the assembled transcripts^11^. The giant panda unaligned transcripts primarily occurred because of incomplete or missing assembly. Many genes were not detected by the Ensembl automated annotation system, resulting in the loss of a large amount of coding information. Therefore, it is necessary to improve the completeness of the panda genome annotation through the analysis of a large-scale transcriptomic reconstruction.

Using the combined transcriptome assembly strategy (reference-based and *de novo* methods), 19,416 expressed novel protein-coding genes were identified from the RNA-seq data from 12 giant panda tissues, including 551 *homology-based genes*, 6,290 *unknown genes*, and 12,575 *hypothetical genes*. Most of these novel protein-coding genes were highly expressed tissue-specific genes (Fig. 3A,B). Highly expressed genes may be more prone to full-length detection with deep-depth and wide-range read coverage. Housekeeping (HK) genes are more conserved than tissue-specific genes during evolution^27^. As a result, the traditional homology search-based method is biased towards the detection of HK genes versus tissue-specific genes, leading to a high false-negative rate for tissue-specific genes. However, expressed tissue-specific genes are more easily detected by RNA-seq data than traditional methods. Proteomic analysis indicated that the novel protein-coding gene identification results were comparable or similar to known gene models. In the proteomic validation of the five selected tissues, the peptides matched 12.62% of the novel protein-coding genes expressed in the five selected tissues, whereas 10.08% of the known gene models expressed in the five selected tissues yielded peptide hits. Therefore, the identification of novel transcripts from the RNA-seq data resulted in the high-quality improvement in giant panda genome annotation from the previous genome annotation methods^6^,^7^,^8^,^9^,^11^. GO annotation analysis showed that some novel protein-coding genes are involved in pigmentation, anatomical structure formation and reproduction, which might be related to the development and evolution of the black-white pelage, adaptive pseudo-thumb and delayed embryonic implantation of the giant panda (Fig. 4). Thus, the identification of these novel protein-coding genes will contribute to future giant panda studies from both structural and functional perspectives.

## Methods

### Ethics statement

Animal care and experiments were conducted according to the guidelines established by the Regulations for the Administration of Affairs Concerning Experimental Animals (Ministry of Science and Technology, China, 2004) and were approved by the Committee for Animal Experiments of the Institute of Zoology, Chinese Academy of Sciences, China. The tissues were sampled immediately after death resulting from diseases.

### Transcriptome library construction and sequencing

Twelve tissue samples were collected from two recently deceased giant panda individuals and immediately stored in liquid nitrogen until RNA extraction. Specifically, the liver, stomach, small intestine, colon, pallium and testis were collected from one male adult, and the pituitary gland, skeletal muscle, tongue, ovary and two skin tissues were collected from one female adult. All samples were sequenced with an Illumina sequencer (San Diego, CA, USA). Both Illumina GAIIx and HiSeq 2000 paired-end DNA libraries were constructed for the 12 tissues using a paired-end sample prep kit (Illumina) according to the standard Illumina Truseq kit Protocol with a fragment size of 500-nt. GAIIx sequencing generated 2 × 80/100-nt paired-end reads, and HiSeq 2000 sequencing generated 2 × 101-nt paired-end reads.

### Extraction of high-quality reads and mapping to the panda genome

High-quality sequencing reads are a prerequisite of good transcriptome assembly. We filtered out low-quality reads, duplicated reads and adaptor contamination reads^28^. Low quality reads were filtered with the following rules: (1) there was an “N” in the first 30-nt in a read; (2) there were more than 3 bases with quality <10 in a read; (3) there were more than 4 bases with quality <13 in a read; (4) the percentage of bases with quality <20 was >60%; and (5) the average quality of all bases was <20. The base quality in a read drops rapidly. The highest wrong base call was observed at the 3′ end of a read, which had implications for the use and interpretation of the Solexa data^29^. The dropping rate was more obvious on read 2 in a pair of reads. As such, we trimmed the read length to 50-nt to gain uniform and high-quality reads for analysis. After quality control, we generated 406.3 million 50-nt paired-end reads, and 38.6 million 50-nt single-end reads for the tissue samples (details shown in Table S1). The reads were mapped to the reference giant panda genome sequence (ailMelv1.0)^2^ by BWA^30^. Mapping results were compared with the current gene models of the panda genome from Ensembl^3^ to characterize the read distribution of the transcribed genome regions.

### Transcriptome reconstruction

Two transcriptome reconstruction methods were used: reference-based assembly by Cufflinks^12^ and *de novo* assembly by Trinity^16^. To cluster fragments into transcripts, the reference-based method detected transcribed fragments covered with mappable reads, constructed a coverage relationship on the basis of the read alignments and read-pair links, and found a minimum path coverage on the directed acyclic graph for the relationship^12^. This method has been directly incorporated into the gene annotation pipeline process in some genome annotations^31^,^32^. The *de novo* method has limitations for the identification of low-abundance transcripts^33^ and the determination of the optimal balance between sensitivity and graph complexity^34^. Hence, the combination of a reference-based method and a *de novo* method is an efficient strategy. For the reference-based method, high-quality paired-end reads of each tissue were mapped to the giant panda reference genome sequence^2^ with TopHat^35^, which is a splice-aware short-read aligner specifically designed for RNA-seq data. Then, cufflinks (a module of Cufflinks) was used to assemble the transcriptome and quantify expression based on the TopHat mapping results from each tissue. Because a typical Trinity assembly required ~500G RAM and thus necessitated a high-memory server to run the pooling assembling, the *de novo* transcriptome reconstruction of the 12 tissues was parallel-processed with Trinity (min_kmer_cov = 3).

### Improvement of genome assembly completeness

Genome assembly completeness can be improved by *de novo* assembly, such as the identification of assembly inconsistency^36^, scaffold extension, and gap closure. The Trinity-assembled transcripts were aligned back to the reference genome by BLAT^17^. The alignment of contiguous transcript segments to multiple scaffolds/contigs indicated that the scaffolds/contigs were adjacent. The optimal alignment of the assembled transcripts on the basis of the BLAT alignment results was detected using the TGNet method^37^. The alignment of contiguous transcript segments to multiple contigs within a scaffold implied a contig connection or partial transcript segments falling into gap regions in a scaffold. Transcripts that confirmed the scaffold connections were used to improve the scaffolding (Fig. 1A) or to identify mis-scaffolding. Potential scaffolding or mis-scaffolding was identified using TGNet^37^. Generally, an ordinal gapped alignment result from segments in a transcript is “++”, and the reverse order of the split-alignment result should be “−−”. Therefore, deviations from this rule in the split-alignment results indicated assembly inconsistencies (Fig. 1B). Additionally, the overlap of genome coordinates of segments in a transcript implied that a nested region existed within the transcript (Fig. 1C). This nesting might be caused by an assembly error in a repeat region. If a transcript covered the scaffold boundary but the partially contiguous segment of this transcript unaligned, then the unaligned transcript segment might fall into a gap between two scaffolds; as a result, a scaffold boundary could be extended (Fig. 1D). If a transcript was located at different contigs within a scaffold but a partial contiguous segment of this transcript unaligned, then the unaligned transcript segment might exactly cover or be adjacent to a gap within the scaffold (Fig. 1E). The last four types of improvements were performed with in-house-writing Perl scripts (Supplementary Note 2–6).

Although scaffold/contig links could be inferred based on the transcript alignment results, the gap length between scaffolds could not be estimated when the intron length of the assembled transcripts was unknown and unavailable from the RNA-seq data. We could provide improved genome sequence regions within only one scaffold. To validate the genome assembly improvement, we downloaded a whole genome sequencing read library of the giant panda (SRR504902) from the SRA database. All paired reads were mapped to the original giant panda genome sequence (ailMelv1.0)^2^ and our improved genome sequence by Bowtie2^38^ (-I 0 -X 1000 -q–no-mixed).

### Identification of candidate novel transcripts

The Trinity-defined transcripts in the 12 tissue transcriptomes were pooled into a transcript set. The transcripts were aligned to the reference genome using BLAT^17^. Transcripts that did not map to a proper genome position were defined as reference-free candidate novel transcripts due to missed genome assembly. The reference-free candidate novel transcripts were clustered by cd-hit^18^ (cd-hit-est module, −c = 0.95, −l = 100, −aS = 0.95, −g = 1) based on sequence similarity. The longest transcript in a cluster was selected as the representative transcript because the longest transcript contained more coding information^10^. Redundant transcripts were filtered out using the sequence clustering results. To remove potential ncRNAs from our assemblies, transcripts that yielded hits to the ncRNA database^19^ were removed. Additionally, transcripts that were successfully aligned to the genome were clustered into transcribed loci based on exon overlaps for transcript features^39^. The longest transcript in a transcribed locus was selected as the representative transcript. Subsequently, all successfully aligned transcript features were converted into the GTF format for cuffcompare^12^ (a module of Cufflinks) analysis. Using cuffcompare, all successfully aligned transcripts were compared with the ailMel v1.62 gene models from Ensembl. Then, the expressed transcripts were tagged as overlapping, matching, or novel where appropriate. From this comparison, transcripts that fell into repeat regions or into known gene regions were filtered out. Expressed transcripts that fell into intergenic regions (and sequence-covered repeat regions, <50%^6^) or pure intron regions or were the antisense transcript of a known gene model were marked as raw reference-dependent candidate novel transcripts. Representative transcripts from these raw reference-dependent candidate novel transcripts were selected as reference-dependent candidate novel genes.

Cufflinks-assembled transcripts with an abundance <1 FPKM were filtered out because these “transcripts” might be the result of transcriptomic noise or a partial assembly. Next, we merged 12 assemblies into a master transcriptome using cuffmerge (a module of Cufflinks) and converted the obtained exon features into the GTF format. Then, the Cufflinks-assembled transcripts were compared with the ailMel v1.62 gene models to identify the reference-dependent candidate novel gene set, as were the Trinity-assembled transcripts.

The two reference-dependent candidate novel gene sets defined by the two assemblers were merged, and repetitive defined genes were identified based on the exon overlaps from the transcript features. For the repetitive defined genes, the longest candidate novel transcripts were retained as the reference-dependent candidate novel genes, and the others were removed. The reference-dependent candidate novel transcripts that had no overlap information were retained directly. After combining the results with the reference-free candidate novel genes defined by Trinity, we obtained a final candidate novel gene set for novel gene validation.

### Novel protein-coding gene validation and gene set annotation analysis

The above-described candidate novel gene sequences were scanned directly for ORFs with Augustus^20^ (alternatives-from-evidence = true, singlestrand = true) to predict potential protein-coding ‘gene’ CDSs with no introns as previously reported^6^,^23^,^40^. The candidate novel genes were predicted independently on each strand because overlapping genes may exist on opposite strands from Illumina sequencing. The predicted ORFs were aligned back to the reference genome using BLAT. The successfully aligned ORF features were converted into the GTF format for cuffcompare analysis. Using cuffcompare, all successfully aligned ORFs were compared to the ailMel v1.62 gene models from Ensembl. ORFs that fell into known gene models or repeat regions were filtered out. The retained ORFs (potential novel protein-coding genes) were used to search the dbEST, nt, and nr databases by BLAST^41^ (−U = F) for homology validation. Full-length novel protein-coding genes were obtained using the following criteria: (1) the predicted ORF should contain basic gene features such as a start codon and a stop codon (otherwise, it was assumed to be a partial gene), and its CDS length should be greater than 150-bp (otherwise, it was assumed to be a small gene)^2^; (2) the e-values of the homology search hits should be ≤1.00E-5, the lengths of the nucleotide alignment segment should be ≥20-nt, and the lengths of the protein alignment segments should be ≥10 amino acids; (3) candidate transcripts that overlap with a known gene model on the opposite strand should have alignment with the ‘+’ strand; and (4) after clustering the independent homology search hits from a target sequence in order, the coverage of the homology target sequence should be ≥95% (sequence coverage = 100*∑((align end − align start − gap size)/target sequence length). After filtering, the ‘novel protein-coding genes’ that had a complete ORF but no homology evidence were defined as *hypothetical genes*^21^. The homology-validated genes were divided into two additional ‘novel protein-coding gene’ groups^21^: *homology-based genes* that were similar to known proteins in the nr database and known cDNA sequences in the nt database and *unknown genes* that were similar to EST sequences in dbEST but had no protein or cDNA homology information. After homology validation, InterProScan^42^ was used to search for functional domains and to predict gene functions of the validated novel protein-coding genes. The resulting GO terms for each gene were attached in InterProScan to annotate functional domains. Visualization of the annotation analysis results of the full-length novel protein-coding gene set was plotted by WEGO^43^.

### Protein extraction and proteome analysis

To verify the reliability of the novel protein-coding gene discovery, we separately analyzed five giant panda tissue proteomes (pallium, pituitary gland, tongue, testis and ovary). A total of 100 μg of each tissue was ground with liquid nitrogen and suspended in 500 μl of lysis buffer (7 M urea, 2 M thiourea, 30 mM Tris, 4% ChAPS, and 1 mM PMSF), followed by 5 min of ultrasonication. After 15 min of centrifugation (12,000 × g, 4 °C), the supernatant protein concentration was measured using the Bradford assay^44^. Proteins with different molecular weights were separated using 12.5% SDS-PAGE electrophoresis. The PAGE gels were cut into different gel slices for separate proteomic analyses according to differences in the protein molecular weight and concentration.

The protein gels were sliced into fragments 2 mm^3^ in size and destained with NH_4_HCO_3_/acetonitrile and an acetonitrile solution. Then, the colorless gel slices were consecutively treated with 10 mM DTT and 55 mM IAM. After the removal of water and vacuum drying, the gel slices were digested overnight with protease; the digestion was terminated by the addition of 10% formic acid. After vacuum drying, 0.1% formic acid was added to obtain a soluble polypeptide solution that was analyzed using a nano-LC-LTQ mass spectrometer (Thermo Finnigan (San Jose, CA, USA)). The mass spectrometric data were analyzed by BioWorks 3.3.1 SP1 (Thermo Scientific) and then searched with the full-length novel protein-coding gene set and the known giant panda gene models (ailMel v1.62) to identify peptide hits. To measure the rate of genes detected by proteomic analysis that were undetected in our identification of novel genes, we also searched peptide hits to the known human (hg19, Ensembl release 62) and dog (canFam3, Ensembl release 62) gene models that were used as common gene models in the Ensembl gene annotation project. After the database search, the peptide hits were filtered with the following parameters: ≥2 distinct peptides, Sp ≥500, RSp ≤5, and xc (± 1, 2, 3, 4) = 1.5, 2.0, 2.5, 3.0.

After peptide filtering, novel gene-expressed (ailMel v1.62) proteins (only the Trinity-defined genes were selected) were identified in the five proteome-validated tissues. Because the Cufflinks-defined novel protein-coding genes were identified from the pooled assemblies, we could not determine in which sample(s) a gene was expressed. The percentage of novel protein-coding genes with proteomic validation was defined as the percentage of expressed genes that contained peptide hits in at least one of the five selected tissues; the percentage of known gene models with proteomic validation was similarly defined. Additionally, the peptides from the known human and dog gene models that yielded peptide hits that did not hit the novel gene sets or the known gene models of giant pandas were also identified; we could evaluate how many genes were missed in our identification on the basis of this analysis.

## Additional Information

**Accession codes**: RNA-seq reads have been deposited in the GenBank/EMBL/DDBJ sequence read archive under accession number SRP063482.

**Data Availability**: The mass spectrometry proteomics data have been deposited in the ProteomeXchange Consortium^45^ via the PRIDE partner repository with the dataset identifiers PXD002917 and 10.6019/PXD002917.

**How to cite this article**: Chen, M. *et al.* Improvement of genome assembly completeness and identification of novel full-length protein-coding genes by RNA-seq in the giant panda genome. *Sci. Rep.* **5**, 18019; doi: 10.1038/srep18019 (2015).

## Supplementary Material

Supplementary Information

Supplementary Information

Supplementary Information

Supplementary Information

Supplementary Information

Supplementary Information

Supplementary Information

## Figures and Tables

**Table 1:** Evaluation of the scaffolding using the Trinity-assembled transcripts.

	**Transcripts**				**Scaffold**	**Connection**
	**++**^1^	**−−**1	**+−/−+**^1^	**Total**		
OK_join^2^	499	2,195	2,083	4,777	1,205	741
OK_merge^3^	56	47	95	198	152	79
PB_merge^4^	605	589	1,269	2,463	987	1,503
Total	1,160	2,831	3,447	7,438	2,106	2,317

^1^The mapping strands of two ordinal, aligned segments resulted in transcripts located across multiple scaffolds, which were oriented ‘+/+’, ‘−/−’, or ‘+/−’.

^2^OK_join: the Trinity-assembled transcript alignment results suggested that these scaffold sequences were adjacent, which was used to improve scaffolding.

^3^OK_merge: the Trinity-assembled transcript alignment results suggested that the most likely situation was that one genomic sequence filled the gap in another.

^4^PB_merge: the Trinity-assembled transcript alignment results suggested that mis-assembly existed within these scaffolds/contigs.

**Table 2:** Proteomic results of five selected tissues (pallium, pituitary gland, tongue, testis and ovary) for the validation of Trinity-defined novel protein-coding genes.

**Group**	**Number of genes with peptide hits**	**Number of candidate novel genes**	**Percent of validated genes**
Homology-based genes	56	342	16.37%
Unknown genes	780	5,407	14.43%
Hypothetical genes	547	5,213	10.49%
Total	1,383	10,962	12.62%
