# Supplementary material for: Improvement of genome assembly completeness and identification of novel full-length protein-coding genes by RNA-seq in the giant panda genome
Source: Sci Rep. 2015 Dec 11;5:18019. doi: 10.1038/srep18019 (PMC4676012; doi:10.1038/srep18019)
Supplement: Supplementary Information [file srep18019-s1.pdf]

Improvement of genome assembly completeness and identification of novel full-length protein-coding genes by RNA-seq in the giant panda genome

Meili Chen[1#], Yibo Hu[2#], Jingxing Liu[1], Qi Wu[2], Chenglin Zhang[3], Jun Yu[1], Jingfa Xiao[1*], Fuwen Wei[2*], Jiayan Wu[1*]

[*] Corresponding author.

E-mail address: xiaojingfa@big.ac.cn (JF XIAO); weifw@ioz.ac.cn (FW WEI); wujy@big.ac.cn (JY WU).

Supplementary Information

Table S1. Read preparation and mapping results.

Tissue	Raw pair reads	High quality reads	Mappable reads	Exon[1]	Exon-junction	Intron	Intergenic[2]	Non-gene Scaffold[3]
Colon	88,516,298	84,917,252	8,472,092	4,146,655	616,132	1,152,047	2,463,892	93,276
Liver	84,511,904	78,130,306	20,414,561	11,259,695	1,044,070	1,237,322	6,091,458	5,441,991
Pallium	78,207,108	73,621,091	61,686,127	20,393,249	3,845,224	7,580,264	25,002,204	4,865,186
Stomach	86,509,228	81,091,816	67,258,738	28,851,664	5,899,847	3,420,672	21,847,876	7,238,679
Ovary	83,640,730	79,008,729	63,073,897	18,346,012	3,661,561	16,446,828	22,638,419	1,981,077
Tongue	86,918,468	81,412,632	48,462,420	18,400,923	3,916,459	6,425,447	18,397,039	1,322,552
Pituitary gland	87,458,546	80,425,690	65,781,666	23,943,886	5,594,655	4,010,804	30,333,761	1,898,560
Small intestine	69,050,518	66,546,946	57,506,869	24,070,896	4,249,016	6,274,928	19,829,359	3,082,670
Testis	83,061,730	79,015,217	66,933,906	21,201,891	4,418,796	6,721,688	21,887,396	12,704,135
Skin 1	80,227,692	76,143,710	61,373,611	20,917,794	4,511,015	8,900,915	24,120,662	2,923,225
Skin 2	51,759,916	49,252,181	8,195,677	3,420,297	611,570	770,642	2,896,709	496,459
Skeletal muscle	23,620,806	21,544,871	15,902,435	7,462,834	1,283,788	1,302,394	4,470,683	1,382,736

1. Reads fall into exon regions including pure exon regions, gene boundaries, and exon-junction.
2. Reads fall into intergenic regions including gene upstream 5,000bp, gene downstream 5,000 bp, and other intergenic regions.
3. Non-gene scaffold meant scaffolds that were not covered by any known gene models.

Table S2.Trinity-assembled transcripts and alignment results.

**Tissue**	**Transcripts**	**Transcribed loci**	**Transcripts**		
			**One scaffold**	**Multiple scaffolds**	**Unaligned**
Colon	26,313	18,946	15,934	10,330	49
Liver	26,512	21,082	17,551	8,909	52
Pallium	81,430	68,919	72,836	7,919	675
Stomach	41,258	38,421	36,621	4,403	234
Ovary	134,622	105,490	121,600	10,166	2,856
Tongue	48,598	41,466	41,047	7,262	289
Pituitary gland	44,748	39,648	41,513	2,817	418
Small intestine	45,197	41,587	39,140	5,907	150
Testis	60,843	54,813	53,327	7,224	292
Skin 1	71,675	59,270	65,351	5,633	691
Skin 2	42,029	36,214	31,718	10,065	246
Skeletal muscle	33,014	29,952	29,679	3,096	239

Table S3. The summary statistics of the original genome assembly and our improved genome assembly.

	**Contig count**	**Contig size (bp)**	**Contig N50 (bp)**	**Contig N90 (bp)**	**Gaps count**
Original genome assembly	200,593	2,245,312,831	39,886	9,848	119,126
Improved genome assembly	197,637	2,246,903,867	41,190	10,081	116,170

Table S4. The comparison results for mapping to the reference of original genome and improved genome.

	Raw read pairs	Mappable pairs			Total mappable pairs	Unmappable pairs
		**Unique mappable pairs**	**Multiple mappable pairs**	**Disconcordant mappable pairs[1]**		
Original genome	77,377,467	65,380,585	4,507,682	27,601	69,915,868	7,461,599
Improved genome	77,377,467	65,364,092	4,528,196	27,579	69,919,867	7,457,600

1. Disconcordant mappable pair meant disconcordant alignment that two reads in a pair were unique mapping, but did not satisfy specified filtering parameters (-I, -X, --fr/--rf/--ff).

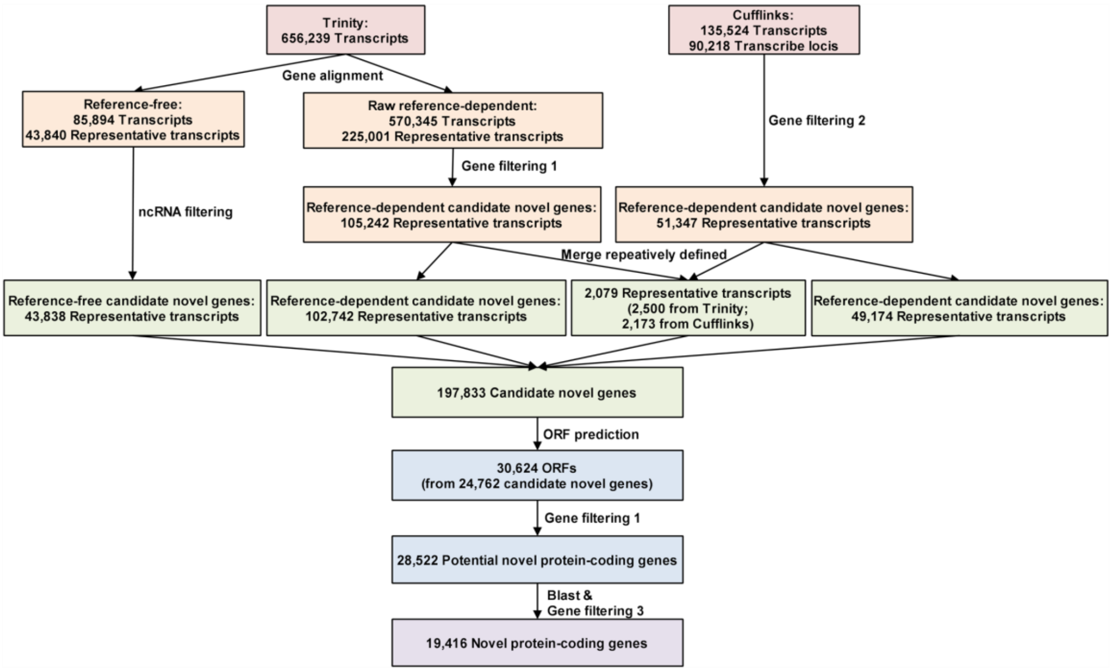

Figure S1. The pipeline for systematic identification of candidate novel protein-coding genes.

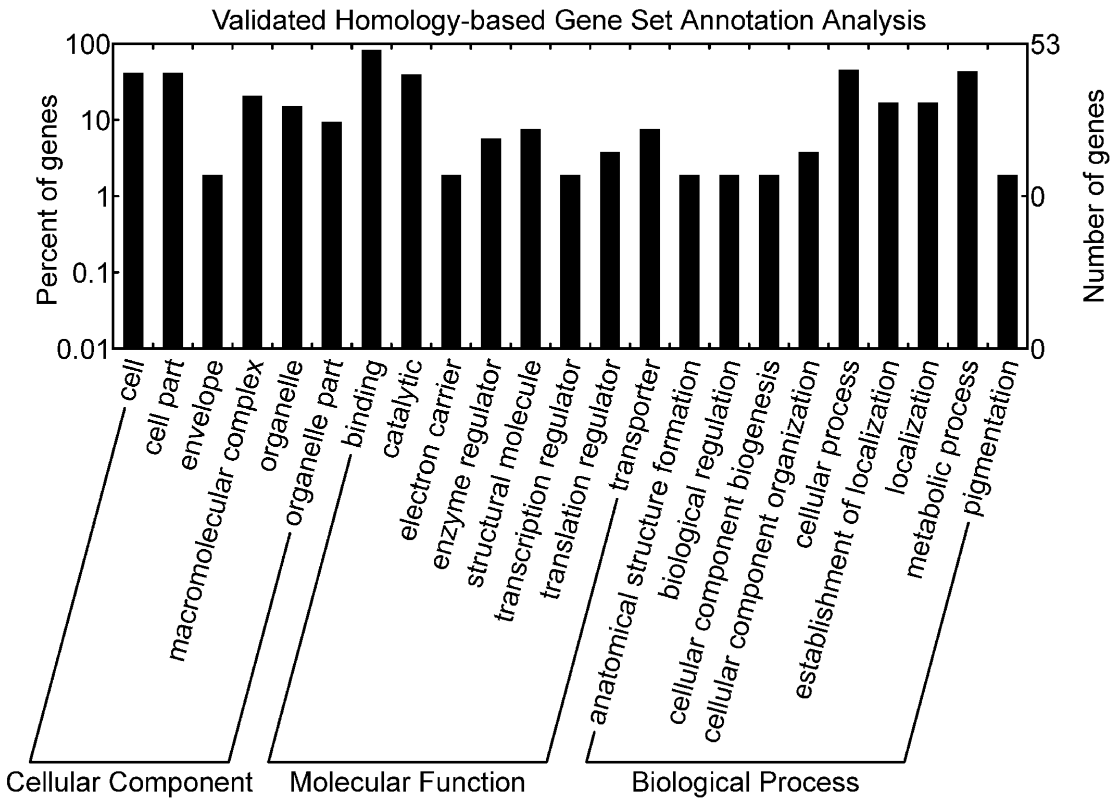

Figure S2. GO functional annotation for homology-based novel genes validated by proteome.
